# Prophylactic radiotherapy, reshaping the immune microenvironment: Effects, mechanisms and clinical applications for preventing tumor metastasis

**DOI:** 10.1016/j.isci.2026.117157

**Published:** 2026-08-11

**Authors:** Jingyuan Liu, Lingling Li, Yuanyuan Yang, Zhen Lan, Xiaohui Zhu, Zhenqiang Sun, Qiming Wang, Yang Liu

**Affiliations:** 1 Department of Radiotherapy, The Affiliated Cancer Hospital of Zhengzhou University & Henan Cancer Hospital, Zhengzhou, China; 2 Department of Colorectal Surgery, The First Affiliated Hospital of Zhengzhou University, Zhengzhou, China; 3 Department of Internal Medicine, The Affiliated Cancer Hospital of Zhengzhou University & Henan Cancer Hospital, Zhengzhou, China

**Keywords:** prophylactic radiotherapy, tumor metastasis, immune microenvironment, pre-metastatic niche

## Abstract

Tumor metastasis remains the leading cause of cancer-related mortality, and effective prevention of clinically occult dissemination remains limited. In the immunotherapy era, the biological role of prophylactic radiotherapy targeting high-risk sites before overt metastasis or regional failure requires critical reassessment. This review summarizes evidence for prophylactic radiotherapy and elective nodal irradiation, distinguishing them from adjuvant radiotherapy and treatment of established but asymptomatic metastases. It further examines the invasion-metastasis cascade, pre-metastatic niche formation, radiation-induced immune microenvironment remodeling, and low-dose hyper-radiosensitivity. Particular attention is given to low-dose radiotherapy’s potential to convert permissive metastatic “soil” into an inhospitable environment, which remains clinically unvalidated. We further discuss major translational barriers, including dose-response windows, toxicity, radiation-induced lymphopenia, biomarker limitations, and integration with immune checkpoint inhibitors. Prophylactic radiotherapy reduces site-specific recurrence but is not universally survival-improving. Future advances require biomarker-enriched trials, lymphocyte-sparing planning, modern imaging surveillance, and mechanistically informed dose and timing selection.

## Graphical abstract

**Figure:**
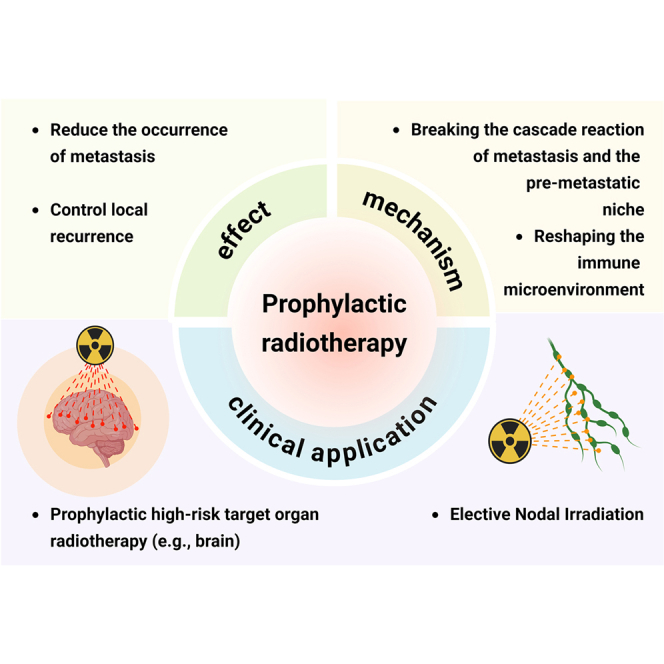

## Teaser

Microenvironment; Biological sciences; Cancer.

## Introduction

Tumor metastasis remains the principal cause of cancer-related death, especially in patients with distant disease, who still experience poor outcomes despite significant advances in systemic treatments.^1^^,^^2^ It is a complex, multi-stage biological process that includes local tissue invasion, entry into the vasculature, survival in circulation, exit into distant tissues, possible dormancy, and eventual establishment of secondary tumors.^3^^,^^4^^,^^5^^,^^6^^,^^7^ Once overt metastasis develops, cure is uncommon for many tumor types. Therefore, an important question in radiation oncology is whether prophylactic irradiation can be applied to selected high-risk organs or lymphatic regions before detectable metastatic progression occurs.

Prophylactic radiotherapy (RT) has historically been based on a cytotoxic concept aimed at eradicating microscopic tumor foci in regions considered at high risk for future recurrence. The most established clinical applications include prophylactic cranial irradiation (PCI) for small-cell lung cancer (SCLC), whole-lung, or prophylactic lung irradiation (WLI/PLI) in selected patients with sarcoma , and elective nodal irradiation (ENI) for certain solid malignancies. In selected clinical settings, these approaches can lower the risk of brain metastases (BMs) , reduce pulmonary metastatic spread, or decrease regional nodal recurrence. However, their impact on overall survival (OS) remains variable and is influenced by factors such as disease context, imaging surveillance strategies, available systemic and salvage therapies, patient selection, and treatment-related toxicity.

Advances in radiobiology have expanded the understanding of prophylactic irradiation beyond its traditional cytotoxic role. Radiation is now recognized as an important modulator of the tumor microenvironment, capable of triggering immunogenic cell death, activating cGAS-STING/type I interferon pathways, enhancing antigen presentation, remodeling myeloid and lymphoid immune populations, and altering stromal and vascular barriers.^8^^,^^9^^,^^10^^,^^11^^,^^12^ In contrast, suboptimal radiation dose, field design, timing, or target selection may lead to adverse effects such as radiation-induced lymphopenia (RIL), injury to draining lymph nodes, activation of transforming growth factor β (TGF-β) and hypoxia-inducible factor-1α (HIF-1α)-mediated pathways, and the development of immunosuppressive or pro-metastatic tissue remodeling.^8^^,^^13^^,^^14^^,^^15^^,^^16^ Thus, the value of prophylactic RT should be assessed not only by its ability to cover anatomical targets, but also by its overall influence on systemic antitumor immunity and organ-specific metastatic microenvironments.

In this review, we present a critical and organized overview of prophylactic RT as a context-specific approach for preventing metastatic disease. We begin by defining its conceptual framework and differentiating true prophylactic irradiation of organs or lymphatic regions from postoperative adjuvant RT, total-body irradiation used for conditioning, and RT directed at established but asymptomatic metastases. We then review the available clinical evidence according to target site and treatment setting, while integrating current understanding of the invasion-metastasis cascade and pre-metastatic niche (PMN) formation. In addition, we examine dose-dependent immune effects, the potential role of low-dose radiotherapy (LDRT), treatment-related toxicities, RIL, biomarker development, combination strategies with systemic therapies, ongoing controversies, and future directions toward precision-based metastasis prevention.

## Prophylactic radiotherapy: Definition and classification

In this review, true prophylactic RT refers to irradiation delivered to a clinically and radiographically disease-free organ or lymphatic region that is considered biologically or anatomically at high risk for future metastatic spread or regional recurrence. A key requirement of this definition is the absence of detectable disease within the irradiated site at the time of treatment. This concept is distinct from several related but fundamentally different radiotherapeutic approaches. First, postoperative adjuvant RT is mainly intended to improve local control by eradicating microscopic residual disease within the primary tumor bed or adjacent regional tissues following surgery. Although preventive in nature, it primarily targets areas of known disease involvement rather than potential future metastatic sites. Second, RT directed at established but asymptomatic metastases, such as high-risk bone lesions, represents early intervention for already existing metastatic disease, rather than true prophylactic treatment of a clinically negative region. ENI lies within a conceptual gray zone. When clinically involved lymph node regions are treated because of predictable patterns of lymphatic dissemination, ENI functions as a form of regional prophylactic RT. However, unlike organ-directed prophylaxis, its immunological effects are more complex because draining lymph nodes serve as critical sites for antigen presentation and T cell activation. As a result, extensive nodal irradiation may eliminate occult tumor deposits while also compromising systemic antitumor immune responses. These conceptual boundaries are outlined in Table 1.

**Table 1:** Conceptual boundaries of prophylactic radiotherapy

Category	Target status	Primary purpose	Examples	Core focus
True prophylactic organ/site radiotherapy	clinically negative but high-risk organ	prevent future metastasis	PCI; PLI/WLI	yes
Regional prophylactic radiotherapy/ENI	clinically negative high-risk nodal basin	prevent regional nodal failure	NPC upper-neck irradiation; RNI	yes, with immune caveats
Postoperative adjuvant radiotherapy	known tumor bed; possible residual disease	improve local/regional control	breast-conserving RT; sarcoma-bed RT	boundary concept
RT for asymptomatic metastases	known metastatic lesion	prevent events/progression	high-risk bone metastasis RT	not true prophylaxis
TBI conditioning	systemic/marrow target	myeloablation before HSCT	TBI before HSCT	boundary concept

ENI, elective nodal irradiation; HSCT, hematopoietic stem cell transplantation; NPC, nasopharyngeal carcinoma; PCI, prophylactic cranial irradiation; PLI, prophylactic lung irradiation; RNI, regional nodal irradiation; RT, radiotherapy; TBI, total body irradiation; WLI, whole lung irradiation.

## Clinical evidence stratified by target site and treatment scenario

Clinical evidence for prophylactic RT is highly heterogeneous across disease sites and treatment contexts. The strongest and most consistent data demonstrate reductions in site-specific metastasis or regional recurrence, while effects on OS remain variable and context-dependent. Accordingly, clinical interpretations should be aligned with the strength of evidence, the endpoints assessed, and the specific disease setting. The major evidence patterns across different clinical scenarios are summarized in Table 2.

**Table 2:** Disease- and site-specific evidence patterns for prophylactic radiotherapy

Disease/context	Common target	Evidence pattern	Key caveat
LS-SCLC	brain	PCI reduces brain metastases and may improve survival in these patients.	potential neurotoxicity should be considered, and patient selection in the MRI era is important.
ES-SCLC	brain	PCI reduces brain metastases, but overall survival benefit is inconsistent.	modern MRI surveillance and immunotherapy may alter the risk-benefit balance.
NSCLC	brain	PCI lowers the incidence of brain metastases.	no consistent overall survival benefit has been demonstrated.
Ewing/osteosarcoma	lung	istorical studies of PLI/WLI suggest improved pulmonary control.	evidence is based on small or older studies, and lung toxicity remains a concern.
NPC	clinically negative neck	ENI shows disease-specific evidence for neck control.	avoid direct extrapolation to other tumor types or nodal regions.
Breast/cervical/esophageal/HNSCC	regional nodes	ENI/RNI improves regional control in selected patient groups.	toxicity, lymphopenia, and interactions with immune checkpoint inhibitors require further evaluation.

ENI, elective nodal irradiation; ES-SCLC, extensive-stage small cell lung cancer; HNSCC, head and neck squamous cell carcinoma; ICI, immune checkpoint inhibitors; LS-SCLC, limited-stage small cell lung cancer; MRI, magnetic resonance imaging; NPC, nasopharyngeal carcinoma; NSCLC, non-small cell lung cancer; PCI, prophylactic cranial irradiation; PLI, prophylactic lung irradiation; RNI, regional nodal irradiation; WLI, whole lung irradiation.

### Brain metastases and prophylactic cranial irradiation

BMs are a common progression site and a major contributor to neurological morbidity in lung cancer, especially SCLC, which frequently spreads intracranially. PCI has the strongest supporting evidence among prophylactic RT approaches. In patients with limited-stage SCLC (LS-SCLC) achieving a complete or partial response following chemoradiotherapy, PCI reduces BM risk by about 50%. Meta-analyses also suggest a modest but meaningful survival benefit, with a ∼5% absolute increase in 3-year OS 5%.^17^^,^^18^ A recent randomized trial further demonstrated lower BM rates with PCI than with MRI surveillance alone. However, no OS was observed, indicating that PCI may offer less advantage with modern MRI monitoring and effective salvage treatments in appropriately selected patients.^19^

The debate is even more prominent in extensive-stage SCLC (ES-SCLC), where outcomes are heavily influenced by baseline MRI use and surveillance intensity. In the EORTC study, PCI reduced symptomatic BMs and improve OS in responders to initial therapy, thereby supporting its clinical use.^20^ However, this trial was limited by the absence of mandatory baseline and serial brain MRI, raising the possibility that some patients had undetected intracranial metastases at enrollment. In contrast, a Japanese randomized trial requiring MRI exclusion of baseline BMs and scheduled MRI surveillance found that PCI reduced BMs without improving OS.^21^ This discrepancy underscores that preventing metastases in a sanctuary site does not necessarily improve survival, particularly in the presence of occult baseline disease, extracranial progression, effective salvage therapy, or treatment-related neurotoxicity.

Evidence from non-small cell lung cancer (NSCLC) likewise underscores the separation between metastasis prevention and survival. In the RTOG 0214 trial of stage III NSCLC, PCI significantly reduced BMs without improving OS.^22^ Subsequent studies have similarly indicated delayed symptomatic intracranial disease and better intracranial control, but no consistent survival benefit.^23^ Notably, the final PRoT-BM study recently reported long-term outcomes in patients with high-risk stage IIIB/IV NSCLC without baseline CNS involvement. In this single-center phase II randomized trial, PCI (25 Gy/10 fractions) significantly reduced 5-year BMs incidence and improved median OS in this high-risk cohort, without clear Mini-Mental State Examination (MMSE) detected neurocognitive decline.^24^ However, the small sample size and single-center design render these results exploratory and in need of validation in larger multicenter phase III trials. Consequently, PCI remains investigational rather than standard practice in NSCLC. Key supporting and opposing evidence across LS-SCLC, ES-SCLC, and NSCLC is summarized in Table 3.

**Table 3:** Clinical evidence and limitations of prophylactic cranial irradiation in lung cancer

Clinical setting	Study	Design/imaging context	Comparison	PCI dose	Brain metastasis outcome	Survival outcome	Interpretation
LS-SCLC	Aupérin meta-analysis^17^	meta-analysis; pre-MRI era	no PCI vs. PCI	Various	58.6% vs. 33.3%	3-year OS 15.3% vs. 20.7%	established PCI benefit; modest OS gain
LS-SCLC	Chen et al.^18^	retrospective; MRI era	surveillance vs. PCI	25–30 Gy/10f	38.5% vs. 28.2%	median OS 32.0 vs. 35.8 months	reduced BM; no significant OS benefit
LS-SCLC	prospective MRI study^19^	prospective randomized; MRI surveillance	MRI surveillance vs. PCI + MRI	25 Gy/10f	37.9% vs. 16.2%	OS 39.9% vs. 43.0%	intracranial control improved; OS unchanged
ES-SCLC	EORTC/NCT00016211^20^	randomized; no mandatory MRI	no PCI vs. PCI	20–30 Gy/10f	40.4% vs. 14.6%	1-year OS 13.3% vs. 27.1%	survival benefit may be confounded by occult BM
ES-SCLC	Japanese trial^21^	randomized; mandatory MRI	observation vs. PCI	25 Gy/10f	63.8% vs. 40.1%	median OS 13.7 vs. 11.6 months	reduced BM; no OS benefit in MRI era
NSCLC	RTOG 0214^22^	randomized; stage III NSCLC	observation vs. PCI	30 Gy/10f	28.3% vs. 16.7%	median OS 2.1 vs. 2.4 years	reduced BM; no OS benefit
NSCLC	NVALT-11/DLCRG-02^23^	randomized; stage III NSCLC	observation vs. PCI	30 Gy/10f	27.2% vs. 7.0%	median OS 21.9 vs. 24.2 months	delayed BM; no OS benefit
High-risk advanced NSCLC	PRoT-BM final analysis^24^	randomized; stage IIIB/IV NSCLC	standard therapy vs. PCI + standard therapy	25 Gy/10f	60-month BM incidence: 78.3% vs. 51.7%	median OS: 19.23 vs. 30.51 months	long-term reduction in BM and possible OS benefit; requires multicenter phase III confirmation
PCI toxicity mitigation	PREMER^25^	randomized	HA-PCI vs. PCI	25 Gy/10f	22.8% vs. 17.7%	median OS 23.4 vs. 24.9 months	HA-PCI may reduce cognitive injury without major efficacy loss

BM, brain metastasis; ES-SCLC, extensive-stage small cell lung cancer; HA-PCI, hippocampal-avoidance prophylactic cranial irradiation; LS-SCLC, limited-stage small cell lung cancer; MRI, magnetic resonance imaging; NSCLC, non-small cell lung cancer; OS, overall survival; PCI, prophylactic cranial irradiation (note: “f” denotes fractions; “Gy” denotes gray).

The benefit of PCI is also limited by concerns over late neurotoxicity. Long-term survivors may develop cognitive decline, memory deficits, fatigue, and reduced quality of life, adversely affecting survivorship. To mitigate these effects, hippocampal-avoidance PCI (HA-PCI) has emerged as a neuroprotective approach that may lessen memory impairment while preserving intracranial control.^25^ Additional strategies, including memantine, improved patient selection, and routine neurocognitive monitoring, may further improve tolerability.

Collectively, PCI highlights both the strengths and the limitations of prophylactic RT. Although it effectively reduces BMs, especially in SCLC, its impact on OS depends on factors such as disease stage, MRI surveillance, extracranial disease burden, salvage treatment availability, and treatment-related toxicity. In the era of advanced imaging and immunotherapy, PCI should therefore be guided by individualized risk-benefit assessment rather than routine use. Key decision factors include baseline MRI findings, expected survival, extracranial disease burden, neurocognitive risk, suitability for hippocampal-sparing techniques, and patient preferences. PCI benefits should also be weighed against intensive MRI surveillance with timely salvage therapy, which may provide a similar balance between efficacy and toxicity in selected patients.

### Pulmonary metastases and lung irradiation: Historical evidence and modern limitations

The lungs are a common site of metastatic spread in several pediatric and young adult sarcomas, particularly Ewing sarcoma and osteosarcoma. As a result, PLI and WLI were historically explored as approaches to eliminate occult pulmonary micrometastases before they became clinically detectable. Much of the evidence supporting these strategies was generated prior to the advent of advanced imaging modalities, modern conformal RT techniques, and current multi-agent chemotherapy regimens, all of which have substantially changed the management of metastatic risk. Early investigations in osteosarcoma produced mixed findings. While one randomized trial evaluating bilateral PLI failed to demonstrate improvements in either OS or the incidence of pulmonary metastases, later retrospective and historical studies reported better pulmonary control and, in some cases, improved disease-free survival. However, these benefits were not consistently accompanied by a clear OS advantage.^26^^,^^27^^,^^28^ Several early studies in Ewing sarcoma also investigated the use of prophylactic or WLI; however, their conclusions were limited by small patient cohorts, retrospective methodologies, and substantial variability in systemic treatment approaches.^29^^,^^30^ Consequently, the historical PLI/WLI literature is best viewed as providing preliminary biological and clinical support for the concept, rather than serving as definitive evidence for its routine application in contemporary practice.^30^

Importantly, the role of WLI in current practice varies according to disease setting. In Ewing sarcoma, it continues to be incorporated into multimodal treatment for carefully selected patients with pulmonary metastatic disease, particularly those with isolated lung metastases or a favorable response to systemic chemotherapy. Nevertheless, the extent of its clinical benefit and the potential for long-term treatment-related toxicity remain areas of ongoing discussion and investigation.^31^^,^^32^ Recent consensus guidelines recommend considering WLI for carefully selected patients with Ewing sarcoma and pulmonary metastases. However, management decisions in the relapsed setting are increasingly tailored to individual patient factors, including metastatic burden, treatment response, age, toxicity, and the availability of local and systemic therapeutic options.^32^^,^^33^ By comparison, the evidence supporting PLI in osteosarcoma remains limited. Contemporary treatment of pulmonary recurrence in osteosarcoma is therefore driven primarily by systemic therapy, close radiologic monitoring, and surgical resection of lung metastases whenever feasible.^26^^,^^27^^,^^28^^,^^30^ Accordingly, PLI/WLI should not be regarded as a universal prophylactic strategy for all sarcomas. Instead, it represents a strategy with historical significance and selective contemporary applicability, with its value largely determined by the specific tumor subtype, pattern of metastatic spread, treatment context, and availability of effective salvage therapies.

Treatment-related toxicity remains a major challenge, as the lungs are particularly sensitive to radiation injury. Potential complications include radiation pneumonitis, pulmonary fibrosis, restrictive lung impairment, and long-term respiratory sequelae, especially in children and adolescents. These risks may be further increased when WLI is combined with intensive systemic treatments, such as high-dose chemotherapy, highlighting the importance of careful patient selection and risk assessment before treatment is undertaken.^34^ Modern RT approaches, including proton-based WLI and intensity-modulated proton therapy (IMPT), have the potential to improve dose conformity and reduce exposure to adjacent critical structures such as the heart and breast. However, current evidence is still limited, largely derived from retrospective analyses, single-center experiences, dosimetric studies, or early-phase reports.^35^^,^^36^ As a result, prospective validation within contemporary multimodality treatment frameworks is necessary before extending PLI/WLI beyond carefully selected sarcoma contexts.

### Elective nodal irradiation for regional metastasis control

ENI aims to treat lymph node regions that are clinically uninvolved but considered at high risk of harboring microscopic disease based on anatomical drainage patterns or biological behavior. In contrast to PCI, which targets a distant sanctuary site, ENI is directed at lymphatic basins that not only serve as potential reservoirs of occult metastases but also play an essential role in immune regulation, including antigen presentation and T cell activation. Accordingly, the role of ENI varies substantially between disease settings and is influenced by multiple factors, including the likelihood of subclinical nodal disease, staging accuracy, risk of distant metastatic spread, normal tissue tolerance, and integration with systemic therapy. A summary of key clinical data evaluating the benefits and limitations of ENI across different tumor types is presented in Table 4.

**Table 4:** Clinical evidence and limitations of elective nodal irradiation across tumor types

Clinical setting	Study	Design/context	Comparison	ENI strategy	Regional-control outcome	Survival/toxicity outcome	Interpretation
N0–N1 nasopharyngeal carcinoma	Tang et al.^37^	multicenter randomized phase III; IMRT era	upper-neck ENI vs. whole-neck ENI	risk-adapted reduction of elective neck volume	3-year regional relapse-free survival: 97.7% vs. 96.3%	similar survival; lower late toxicity with upper-neck irradiation	selective ENI can preserve regional control while reducing toxicity in low-risk neck regions.
Head-and-neck tumors in immunotherapy context	Darragh et al.^38^; Marciscano et al.^39^	preclinical and translational immune studies	tumor-only RT vs. RT + ENI	inclusion of draining lymph nodes	ENI may reduce occult nodal relapse in principle	ENI attenuated antigen-specific T cell responses and RT-immunotherapy efficacy	in the immunotherapy era, lymph-node-sparing approaches may better preserve systemic antitumor immunity.
High-risk early-stage breast cancer	MA.20/Whelan et al.^40^	randomized phase III	whole-breast irradiation vs. whole-breast + RNI	internal mammary, supraclavicular, and axillary nodal irradiation	reduced regional and distant recurrence	no significant OS benefit; increased pneumonitis and lymphedema	RNI improves disease control in selected high-risk patients but adds morbidity.
Stage I-III breast cancer	EORTC 22922/10925/Poortmans et al.^41^	randomized phase III; 15-year follow-up	no IM-MS irradiation vs. IM-MS irradiation	internal mammary and medial supraclavicular irradiation	reduced breast cancer recurrence	reduced breast cancer mortality; no significant OS improvement	supports nodal irradiation as a recurrence-reducing strategy rather than a universally OS-improving intervention.
Locally advanced NSCLC	Chen et al.^42^; Li et al.^43^	prospective randomized study and meta-analysis	IFRT vs. ENI	omission of clinically uninvolved nodal stations	no clear increase in elective nodal failure with IFRT	IFRT may reduce toxicity and facilitate dose escalation	routine ENI is generally not favored in modern NSCLC radiotherapy.
Esophageal cancer	Liu et al.^44^; Chen et al.^45^	network meta-analysis and modern retrospective analysis	IFI vs. ENI	involved-field versus elective nodal coverage	ENI may reduce regional microscopic disease, but results are heterogeneous	no consistent survival advantage; larger fields may increase toxicity and lymphocyte exposure	ENI remains controversial and should be individualized by tumor location, histology, and treatment intent.
High-risk cervical cancer	RTOG 79–20/Rotman et al.^46^; Wang et al.^47^; Soetomo et al.^48^	historical randomized trial, modern review, and RCT meta-analysis	pelvic RT vs. pelvic + para-aortic RT	prophylactic para-aortic irradiation	potential reduction in para-aortic nodal metastases	historical OS signal with increased severe toxicity; recent randomized-trial meta-analysis found no clear OS improvement	extended-field prophylaxis may be considered in carefully selected high-risk patients, but modern routine use requires caution.

ENI, elective nodal irradiation; IFI, involved-field irradiation; IFRT, involved-field radiotherapy; IM-MS, internal mammary and medial supraclavicular; IMRT, intensity-modulated radiotherapy; NSCLC, non-small cell lung cancer; OS, overall survival; RCT, randomized controlled trial; RNI, regional nodal irradiation; RT, radiotherapy.

In nasopharyngeal carcinoma (NPC), ENI is strongly justified by the characteristic and highly predictable pattern of cervical lymphatic dissemination. Nevertheless, contemporary data increasingly support a shift from indiscriminate nodal coverage toward more risk-adapted, de-escalated irradiation strategies. In a multicenter phase III randomized trial involving patients with N0–N1 NPC, upper-neck-only elective irradiation demonstrated non-inferiority to whole-neck irradiation for regional relapse-free survival while also reducing long-term treatment-related toxicity.^37^ This study is especially significant as it highlights that modern ENI is not about expanding elective fields as much as possible, but rather about maintaining effective regional disease control while minimizing unnecessary irradiation of normal tissue.

In breast cancer, regional nodal irradiation (RNI) represents one of the most well-established ENI-related approaches with robust clinical evidence. The MA.20 trial demonstrated that the addition of RNI to whole-breast irradiation reduced the risk of disease recurrence; however, it did not lead to a significant improvement in OS and was associated with higher rates of toxicities such as pneumonitis and lymphedema.^40^ Similarly, the EORTC 22922/10925 study showed that irradiation of the internal mammary and medial supraclavicular lymph nodes decreased both recurrence rates and breast cancer-specific mortality, although a statistically significant improvement in OS was not observed at long-term follow-up.^41^ Collectively, these findings support the use of RNI in carefully selected high-risk patients, while also emphasizing that gains in regional control or disease-free survival do not always translate into a consistent OS advantage.

In locally advanced NSCLC, the role of ENI has moved toward more selective use. The adoption of PET/CT-based staging, concurrent chemotherapy, and advanced conformal radiation techniques has led to the widespread replacement of routine ENI with involved-field radiotherapy (IFRT). A prospective randomized study showed that IFRT did not result in higher rates of isolated nodal failure and allowed safer dose escalation compared with ENI, while a subsequent meta-analysis similarly reported no significant difference in elective nodal failure between the two approaches.^42^^,^^43^ These data do not support the routine use of ENI in NSCLC, particularly given that larger radiation fields may increase lung and esophageal toxicity and potentially impair systemic immune function.

In esophageal cancer, the use of ENI remains a subject of ongoing debate. Although the esophagus has an extensive longitudinal lymphatic network that supports the rationale for elective nodal coverage, existing studies are highly variable, differing in histological subtype, tumor location, treatment strategy, and surgical management. In the neoadjuvant setting, a network meta-analysis reported no significant differences between ENI-based and involved-field irradiation approaches in terms of OS, locoregional recurrence, distant metastasis, R0 resection rates, or postoperative mortality.^44^ More recent retrospective studies in locally advanced esophageal squamous cell carcinoma suggest that ENI may expose surrounding organs at risk to higher radiation doses and be associated with increased treatment-related toxicity, whereas IFI can yield comparable disease-free and OS outcomes in appropriately selected patients.^45^ Accordingly, ENI in esophageal cancer should be tailored to individual risk profiles rather than presumed superior solely on the basis of more extensive target coverage.

In cervical cancer, prophylactic para-aortic irradiation is considered a further borderline indication. The RTOG 79–20 trial reported an improvement in 10-year OS with extended-field (pelvic plus para-aortic) irradiation compared with pelvic irradiation alone; however, this benefit was accompanied by significantly higher rates of severe toxicity. Importantly, these results were generated in an era prior to contemporary standards such as concurrent chemoradiotherapy, IMRT-based planning, PET-based staging, and modern image-guided brachytherapy.^46^ Contemporary reviews indicate that prophylactic extended-field irradiation may be technically feasible using conformal or IMRT-based techniques in carefully selected high-risk patients; however, its impact on OS remains unclear.^47^ A recent meta-analysis of randomized studies suggested a potential reduction in para-aortic nodal failure with prophylactic para-aortic irradiation, but no definitive improvement in OS, underscoring the importance of individualized patient selection rather than routine application of extended-field treatment.^48^ Accordingly, prophylactic para-aortic irradiation should be considered a risk-adapted approach rather than a standard practice for all patients.

The advent of immunotherapy introduces additional complexity to the role of ENI. Regional lymph nodes serve not only as potential sites of occult microscopic disease but also as critical hubs for dendritic cell migration, T cell priming, clonal expansion, and epitope spreading. Preclinical and translational studies show that ENI may impair systemic antitumor immune responses and potentially diminish the efficacy of RT combined with immune checkpoint inhibitors (ICIs) by reducing antigen-specific CD8^+^ T cell activation, trafficking, and effector function.^38^^,^^39^ More recent studies indicate that both the timing and dose distribution to tumor-draining or clinically uninvolved lymph nodes may significantly influence treatment outcomes. In particular, delayed irradiation of nodal regions may help preserve the effectiveness of radio-immunotherapy combinations, whereas greater dose exposure to lymph node volumes has been linked to reduced T cell activation and less favorable clinical outcomes.^49^^,^^50^ Accordingly, when systemic immune stimulation is a major therapeutic objective, strategies such as involved-field irradiation, sentinel-node-guided targeting, delayed nodal treatment, or lymph-node-sparing strategies may provide a more favorable therapeutic balance than comprehensive ENI.

### Adjacent concepts: Postoperative adjuvant radiotherapy, total body irradiation, and RT for asymptomatic metastases

Several established applications of RT have a preventive component but do not meet the strict definition of prophylactic RT adopted in this review. As summarized in Table 1, these approaches differ from true prophylactic irradiation of clinically negative organs or sites with respect to target characteristics, treatment objectives, and the underlying evidence supporting their use. Postoperative adjuvant RT is administered to the primary tumor bed or surrounding regional tissues that may harbor microscopic residual disease after surgery. Its main objective is to improve local and regional disease control rather than to prevent future metastatic seeding in a clinically uninvolved organ or nodal basin. For instance, the 10-year results of the PRIME II trial showed that omitting whole-breast irradiation in selected older patients with low-risk early breast cancer led to a higher rate of local recurrence, while distant recurrence and OS remained unchanged.^51^ Likewise, RT delivered to established but asymptomatic metastatic lesions, such as high-risk bone metastases, should be regarded as early intervention for known metastatic disease rather than prophylactic treatment of a disease-free site.^52^ Total body irradiation (TBI) used as conditioning before hematopoietic stem-cell transplantation is also fundamentally different, as its purpose is systemic myeloablation and immunosuppression rather than targeted prevention of metastasis in a specific organ or anatomical region.^53^^,^^54^

For this reason, these modalities are not discussed in detail in the present review. They are included solely to define the conceptual boundaries of prophylactic RT and to distinguish true irradiation of clinically negative but high-risk metastatic or nodal sites from postoperative adjuvant treatment, early intervention for known metastatic lesions, and systemic conditioning approaches.

## Biological rationale for prophylactic radiotherapy: Targeting the metastatic process and its microenvironment

**Figure 1:**
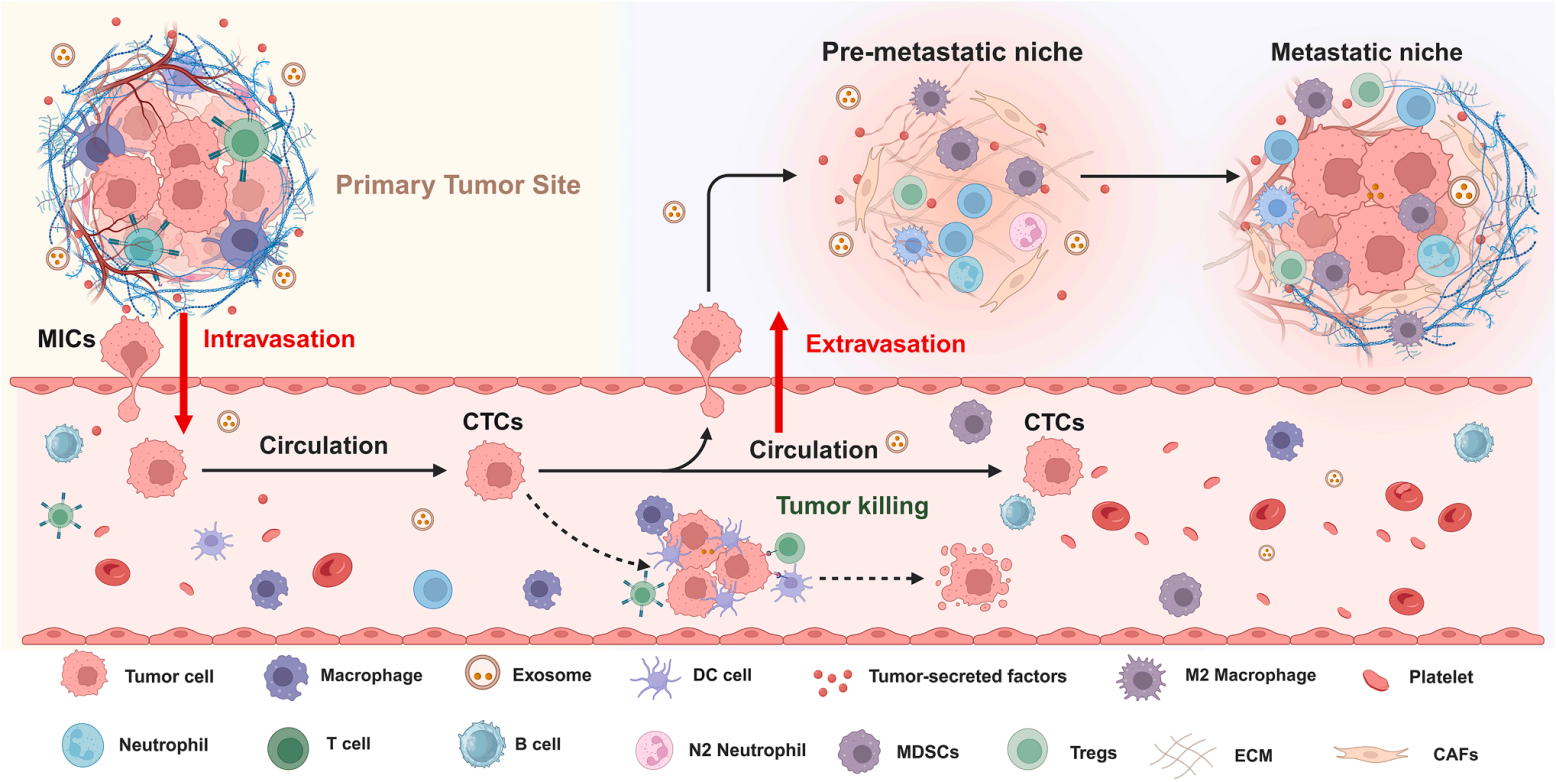
Tumor invasion-metastasis cascade and pre-metastatic niche formation Primary tumors generate metastasis-initiating cells that undergo local invasion, intravasation, survival as CTCs, extravasation, and colonization of distant organs. Meanwhile, tumor-derived soluble factors and extracellular vesicles precondition distant sites by recruiting immunosuppressive and stromal cells, including myeloid-derived suppressor cells (MDSCs), regulatory T cells (Tregs), M2 macrophages, N2 neutrophils, and cancer-associated fibroblasts (CAFs), and promoting extracellular matrix remodeling. This evolution from a pre-metastatic to a metastatic niche underpins prophylactic radiotherapy, aimed at eliminating occult tumor “seeds” or modifying the permissive metastatic “soil.”

Metastasis is a complex, multistep process governed by interactions among evolving tumor cells, the host immune system, stromal components, and the unique biological features of distant organs. The metastatic cascade encompasses a series of events, including local tumor invasion, intravasation, survival of circulating tumor cells (CTCs), extravasation into distant tissues, persistence of disseminated tumor cells (DTCs) in a dormant state, and ultimately the establishment of overt metastatic lesions. Despite entering the circulation or reaching distant tissues, the majority of CTCs and DTCs do not successfully establish metastases. This observation suggests that metastatic progression is determined not only by the biological properties of the tumor “seed” but also by the suitability of the target-organ “soil.” Recent research indicates that metastasis is a highly context-dependent process but varies across disease stages and organ sites rather than following a simple linear process. Factors such as immune surveillance, vascular characteristics, extracellular matrix remodeling, and stromal interactions play a critical role in determining whether DTCs remain dormant or develop into clinically detectable metastases.^6^^,^^55^^,^^56^ The major steps of the invasion-metastasis cascade, together with the evolution from a PMN to an established metastatic niche, are illustrated in Figure 1.

The PMN represents a critical biological bridge between occult tumor dissemination and potential preventive interventions. Even before tumor cells reach distant organs, primary tumors can modify remote tissues by releasing cytokines, chemokines, soluble mediators, metabolites, and extracellular vesicles. These signals promote the recruitment of bone marrow-derived cells, alter macrophages and neutrophil phenotypes, expand immunosuppressive cell populations such as myeloid-derived suppressor cells and regulatory T cells, remodel the extracellular matrix, increase vascular permeability, and weaken local antitumor immunity. Collectively, these changes create a microenvironment that favors CTC retention, DTC persistence, immune evasion, and ultimately the establishment of metastatic lesions.^56^^,^^57^

This biological framework provides a compelling rationale for prophylactic RT while also highlighting its inherent limitations. In a prophylactic setting, the irradiation site is clinically and radiographically free of detectable disease. Consequently, the goal is not to eradicate established tumors but to reduce the risk of future metastatic or regional recurrence by targeting occult tumor cells, eliminating microscopic deposits, or modifying a microenvironment that has been conditioned to support metastatic growth. Conventional-dose prophylactic RT is thought to exert its effects mainly through the eradication of microscopic tumor foci via DNA damage and the induction of immunogenic cell death. In contrast, LDRT has been proposed to function primarily as a regulator of the local microenvironment, influencing processes such as macrophage polarization, myeloid-cell activity, dendritic-cell activation, T cell recruitment, vascular permeability, and stromal remodeling.^11^^,^^12^^,^^58^^,^^59^ Conversely, suboptimal target selection, overly large treatment fields, or excessive radiation doses may compromise antitumor immune surveillance. Potential adverse effects include irradiation of tumor-draining lymph nodes that are essential for T cell priming, increased RIL, and activation of biological pathways associated with metastatic progression, including TGF-β/HIF-1α signaling, epithelial-mesenchymal transition (EMT), and vascular remodeling.^13^^,^^14^^,^^15^^,^^16^^,^^39^^,^^59^^,^^60^

Therefore, the metastatic cascade and PMN concept together define potential points of intervention for prophylactic RT, including eradication of occult tumor “seeds,” modulation of a permissive metastatic “soil,” or, in certain situations, unintended disruption of immune and stromal components that are important for controlling metastatic spread. This perspective also helps explain why prophylactic RT cannot be universally applied across all tumor types or anatomical sites. Variations in metastatic tropism, immune microenvironment, vascular architecture, lymphatic drainage patterns, and tissue radiosensitivity collectively account for differing clinical roles of PCI, PLI/WLI, and ENI. These biological and anatomical differences also contribute to the key challenges related to dose selection, treatment timing, toxicity, and biomarker development, which are discussed in the following sections.

## Dose-dependent mechanisms of prophylactic radiotherapy

The biological impact of prophylactic RT depends on fraction size, total dose, irradiated volume, target organ, timing with systemic therapy, and baseline immune status. It should therefore not be viewed merely as low-dose definitive RT, but as a biological continuum with context-dependent effects. Conventional doses may eliminate occult tumor cells and induce immunogenic signaling, whereas LDRT primarily reshapes immune and stromal components of the microenvironment. Higher doses or large fields may be harmful, causing lymphocyte depletion, vascular damage, and immunosuppressive tissue remodeling.^8^^,^^9^^,^^11^^,^^58^^,^^59^^,^^60^ Therefore, the shift between immune stimulation and suppression is better seen as a context-dependent biological window rather than a fixed dose cutoff. This window likely varies across organs and settings due to differences in radiosensitivity, vascular structure, immune composition, and lymph-node function in sites such as brain, lung, liver, bone, and lymphatic regions.

### Direct cytotoxicity, immunogenic cell death, and cGAS-STING signaling

Conventional prophylactic RT can eliminate microscopic tumor deposits through multiple mechanisms, including DNA double-strand breaks, reactive oxygen species-mediated damage, cell-cycle arrest, mitotic catastrophe, apoptosis, and necrotic cell death. Although prophylactic dosing is typically lower than that used for overt microscopic disease, it may still be adequate to eliminate or significantly reduce radiosensitive occult tumor cells or micrometastatic clusters, particularly in the setting of low tumor burden. Beyond its direct cell-killing effects, RT can also convert tumor-cell death into an immunogenic process. Radiation-induced immunogenic cell death is characterized by exposure of calreticulin on the cell surface, release of ATP and HMGB1, and the presentation of tumor-associated antigens and neoantigens. Collectively, these danger signals promote dendritic-cell maturation, enhance antigen cross-presentation, and drive activation of cytotoxic T-cells.^8^^,^^10^^,^^58^

**Figure 2:**
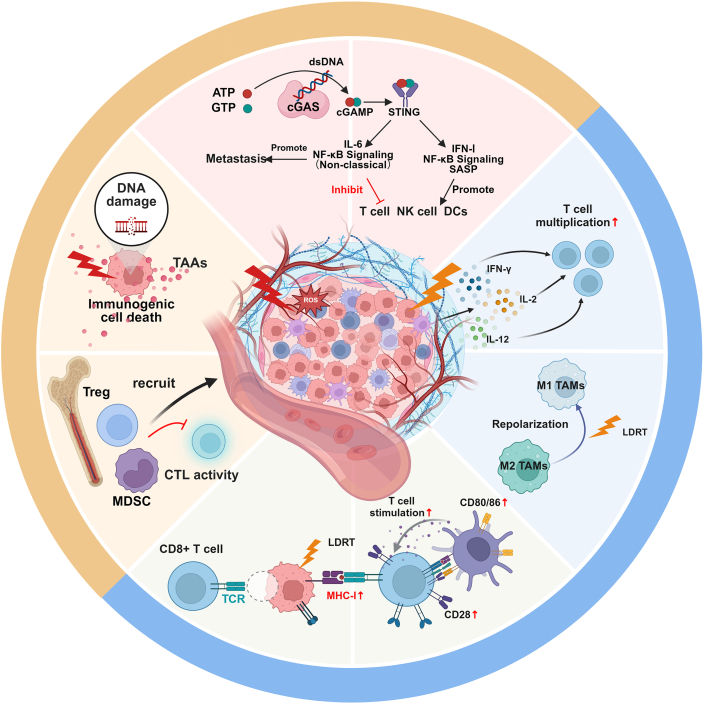
Dose-dependent immune and stromal effects of radiotherapy Conventional prophylactic radiotherapy can eradicate occult tumor cells via DNA damage and induce immunogenic cell death, promoting antigens, and activating cGAS-STING/type I interferon signaling. LDRT instead acts primarily through immune-stromal reprogramming, including macrophage repolarization, improved antigen presentation, enhanced T- and NK-cell infiltration, reduced Tregs and MDSCs, and vascular normalization. Conversely, high doses, large fields, or suboptimal timing may cause lymphopenia, lymphatic injury, and immunosuppressive remodeling via TGF-β and HIF-1α, with increased myeloid recruitment. Collectively, these effects define a context-dependent therapeutic window for prophylactic radiotherapy.

In parallel, radiation can generate cytosolic DNA fragments and micronuclei that activate the cGAS-STING pathway, leading to type I interferon signaling and subsequent recruitment of antigen-presenting cells and effector lymphocytes.^10^ Together, these effects support a broader view of prophylactic RT as more than simple anatomical eradication; in appropriately selected contexts, it may also strengthen immune recognition and clearance of otherwise clinically occult tumor deposits. However, this pathway is highly dependent on both dose and treatment context. High single-fraction doses may upregulate DNA exonucleases such as TREX1, thereby reducing cytosolic DNA accumulation and weakening STING-mediated immune activation.^61^ In addition, large irradiation fields can significantly expose circulating lymphocytes, draining lymph nodes, and hematopoietic tissues, ultimately diminishing the immune capacity required for effective systemic antitumor responses.^13^^,^^38^^,^^39^^,^^49^^,^^50^^,^^59^ As a result, immune activation after RT is not guaranteed and is contingent on preserving adequate immune priming and effector function within the treatment plan. These dose-dependent cytotoxic, immunomodulatory, and immunosuppressive effects are illustrated in Figure 2.

### LDRT-mediated immune and stromal reprogramming

LDRT is commonly defined as delivery of approximately 0.5–2 Gy per fraction with a relatively low total cumulative dose, although exact definitions vary across the literature. In contrast to conventional or ablative RT, LDRT is unlikely to eradicate established tumor masses through direct cytotoxic mechanisms. Instead, its potential therapeutic relevance is primarily attributed to modulation of the immune and stromal microenvironment. Preclinical evidence suggests that LDRT can enhance antigen presentation, increase expression of MHC-I and co-stimulatory molecules, stimulate dendritic cell function, promote recruitment and trafficking of T-cells and natural killer (NK)-cells, and reprogram macrophages from an immunosuppressive M2-like state toward a more pro-inflammatory M1-like phenotype.^11^^,^^12^^,^^58^^,^^60^

LDRT may further alleviate immunosuppressive conditions within the tumor microenvironment. Studies have shown that it can diminish the suppressive activity of Tregs and MDSCs, increase the ratio of CD8^+^ T cell to Tregs, enhance NK cell cytotoxicity through mechanisms including NKG2D, and improve endothelial adhesion molecules that promote trafficking into tumors.^11^^,^^12^ In addition, LDRT may also exert important effects on stromal and vascular components of the tumor microenvironment. By reducing aberrant vascular leakage, regulating fibroblast activity, and altering extracellular matrix remodeling, LDRT may promote the transition of immunologically cold tumors toward a more inflamed and immune-supportive state.^9^^,^^11^^,^^58^^,^^60^^,^^62^

**Figure 3:**
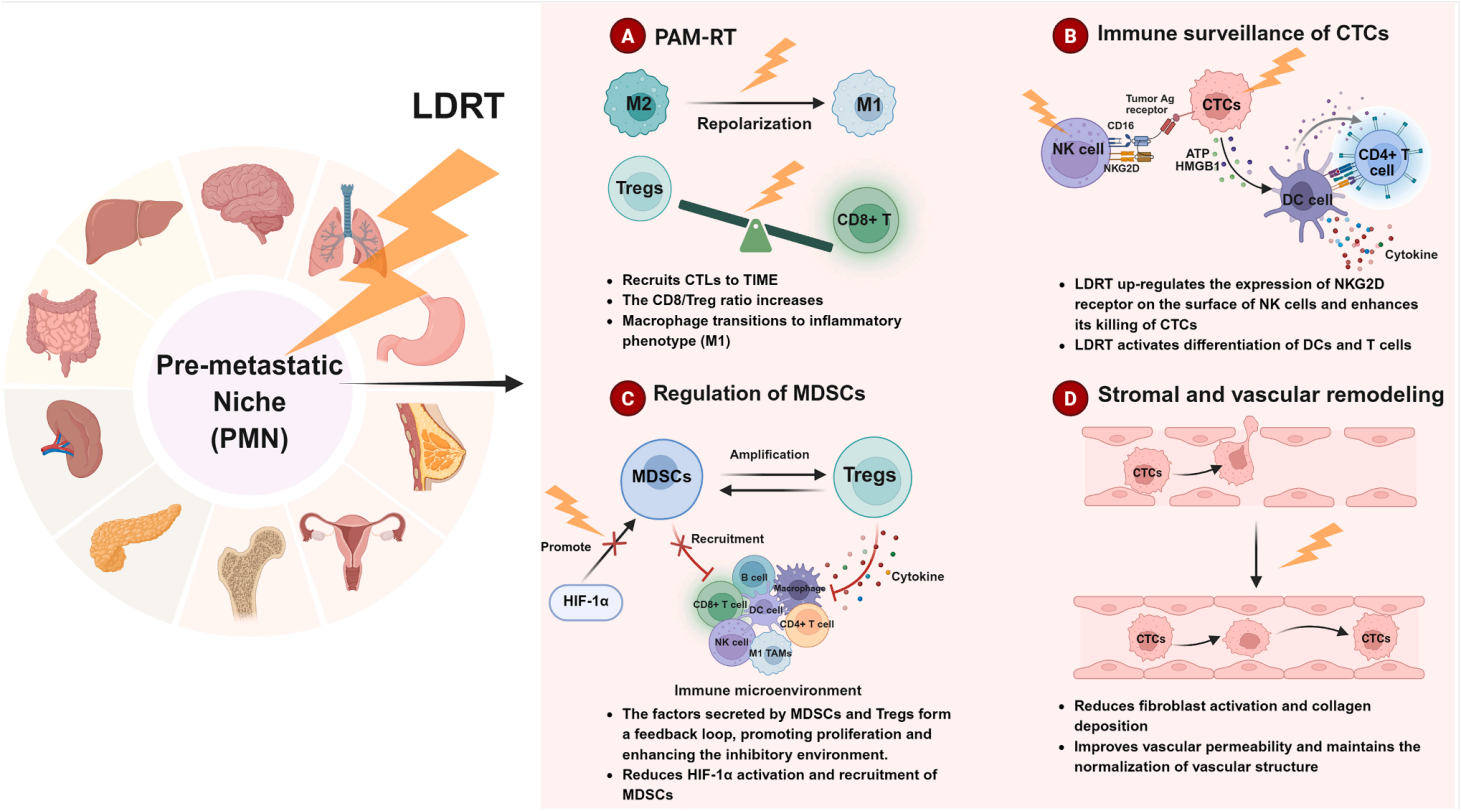
Hypothetical mechanisms of PMN-directed prophylactic LDRT Primary tumors can prime distant organs via soluble mediators and extracellular vesicles, promoting myeloid recruitment, Treg and MDSC accumulation, macrophage polarization, vascular dysfunction, extracellular matrix remodeling, and immune suppression. Prophylactic LDRT may partially reverse these PMN changes by activating dendritic cells, enhancing CD8^+^ T cell and NK-cell activity, promoting M1-like macrophage repolarization, reducing immunosuppressive populations, and restoring vascular-stromal homeostasis. This model is largely preclinical and requires validation in prospective clinical studies.

Within a PMN framework, these effects may be particularly important. Primary tumors are known to prepare distant tissues for metastatic colonization by promoting myeloid-cell recruitment, dampening local immune responses, increasing vascular permeability, and restructuring the extracellular matrix.^56^^,^^57^ By counteracting these processes, LDRT may theoretically render susceptible organs less supportive of CTC seeding, DTC persistence, and subsequent metastatic expansion. For instance, preclinical studies have shown that low-dose irradiation of the lungs following treatment of the primary tumor can reduce metastatic burden while enhancing antitumor immunity. Reported effects include increased infiltration of CD8^+^ T-cells, repolarization of macrophages toward a more immune-supportive phenotype, and decreased accumulation of Tregs or MDSCs.^11^^,^^12^^,^^58^^,^^60^ Nevertheless, evidence supporting PMN-targeted LDRT is currently derived largely from experimental models. Critical parameters, including optimal dose, fractionation schedule, treatment volume, timing of administration, and patient-selection strategies, have yet to be established. Consequently, prophylactic LDRT should presently be regarded as an investigational immunomodulatory intervention rather than a validated clinical strategy. A hypothetical model of PMN-directed prophylactic LDRT is shown in Figure 3.

### Low-dose hyper-radiosensitivity and micrometastatic disease

A further biological basis for prophylactic low-dose irradiation is the phenomenon of low-dose hyper-radiosensitivity (HRS). HRS refers to the observation that some cell populations exhibit disproportionately high sensitivity to radiation at very low doses, typically between 0.1 and 0.5 Gy, before transitioning to a more radioresistant state as the dose increases. This effect is thought to result, at least in part, from inadequate activation of DNA damage recognition and repair pathways at low radiation exposures.^63^^,^^64^

HRS may be particularly relevant in the setting of micrometastatic disease, as occult metastatic deposits differ biologically from established tumors. These lesions are frequently small, poorly vascularized, and composed of slowly proliferating or dormant cells. Experimental evidence suggests that such quiescent micrometastatic populations may exhibit increased sensitivity to very low radiation doses, potentially offering a window for early therapeutic intervention.^63^^,^^64^ Despite its biological plausibility, the relevance of HRS to prophylactic RT remains largely unvalidated in clinical settings. The susceptibility of micrometastatic lesions to low-dose radiation is likely influenced by multiple factors, including tumor histology, proliferative status, DNA repair competence, the surrounding immune microenvironment, and characteristics of the host organ niche. Therefore, HRS should be regarded as a theoretical mechanism that may support the rationale for prophylactic LDRT rather than as proof of clinical benefits.

### Potential pro-metastatic and immunosuppressive effects of RT

While RT can elicit potent antitumor effects, the biological responses it induces may have unintended consequences when applied prophylactically. For example, radiation-triggered activation of TGF-β can stimulate fibroblast activity, enhance collagen accumulation, promote extracellular matrix remodeling, induce EMT-associated programs, and contribute to immune suppression.^14^ Similarly, stabilization of HIF-1α in hypoxic or radiation-damaged tissues may upregulate vascular endothelial growth factor (VEGF), enhance angiogenic signaling, recruit myeloid cells, and potentially facilitate metastatic progression.^15^ Emerging evidence further indicates that radiation-induced amphiregulin signaling may contribute to myeloid-cell reprogramming and, under certain conditions, support metastatic progression.^16^ Radiation may also cause a temporary increase in vascular permeability, which can facilitate immune-cell trafficking, but under certain adverse biological conditions, it could support tumor cell extravasation.

The effect of RT on myeloid and lymphoid populations is also highly relevant. Irradiation can promote the recruitment and expansion of MDSCs through mechanisms involving CSF1/CSF1R signaling, CCR2-dependent pathways, inflammatory mediators, and context-specific activation of the cGAS-STING axis. These MDSCs can subsequently enhance Treg accumulation, inhibit CD8^+^ T cell function and NK-cell function, and contribute to the maintenance of an immunosuppressive PMN.^11^^,^^14^^,^^16^ Extensive radiation fields may additionally induce radiation-associated lymphopenia by depleting circulating lymphocyte populations and disrupting lymph-node structures that are critical for antigen presentation and T cell activation.^13^^,^^38^^,^^39^^,^^49^^,^^50^^,^^59^^,^^65^ These consequences are of particular concern when prophylactic RT is administered alongside immune checkpoint blockade, as the efficacy of such therapies depends on the preservation of systemic immune competence.

Taken together, the impact of prophylactic RT on metastatic progression is likely to be highly context-dependent and may exert both beneficial and detrimental effects. Optimized treatment parameters, including dose, fractionation schedule, target volume, and timing, may help eradicate occult disease and remodel permissive PMNs. Conversely, suboptimal treatment approaches could compromise immune surveillance or induce stromal alterations that facilitate metastatic dissemination. Future development of prophylactic RT approaches should prioritize minimization of irradiated tissue volumes, preservation of lymphocyte-rich structures, organ-specific risk assessment, and prospective validation using biomarker-guided strategies.

## Strategic dose selection and organ-specific risk adaptation

Dose selection should be guided by the desired biological effect rather than predefined numerical dose thresholds. When the goal is eradication of occult tumor deposits within high-risk anatomical sites, conventional prophylactic radiation doses may be justified. Conversely, if the objective is to modulate a permissive PMN or augment responsiveness to immunotherapy, LDRT is biologically appealing but remains experimental. When the objective is eradication of established oligometastatic lesions, stereotactic body RT (SBRT) or stereotactic radiosurgery (SRS) should be classified as therapeutic interventions rather than prophylactic measures. Accordingly, these modalities represent fundamentally distinct biological strategies rather than simply variations in radiation dose.^11^^,^^12^^,^^58^^,^^59^^,^^60^

Equally critical, the immunological and toxicity landscape varies according to organ and irradiated volume. In the brain, concerns regarding neurocognitive impairment and hippocampal exposure restrict the feasibility of PCI.^25^ In contrast, in the lung, the risk of radiation pneumonitis and long-term decline in pulmonary function limits the use of whole-organ or extensive-field irradiation.^34^ In nodal irradiation, the primary balance lies between eradication of occult nodal disease and preservation of lymph-node-mediated immune function.^38^^,^^39^^,^^49^^,^^50^ However, irradiation of marrow-rich or large fields shifts the risk profile toward RIL and hematopoietic toxicity.^13^ These key considerations are summarized in Table 5.

**Table 5:** Dose strategy, biological purpose, and organ-specific considerations for prophylactic radiotherapy

Dose strategy	Typical range	Dominant biological objective	Potential immune/stromal effect	Major risk	Best-fit context	Clinical readiness
LDRT	∼0.5–2 Gy/fraction; low cumulative dose	immune and stromal reprogramming rather than direct tumor sterilization	macrophage M1-like polarization, reduced MDSC/Treg suppression, improved antigen presentation, vascular normalization	insufficient cytotoxicity; uncertain durability; context-dependent immune suppression	investigational PMN modulation; possible ICI sensitization	preclinical or early translational; not standard prophylaxis
Conventional prophylactic radiotherapy	∼1.8–2.2 Gy/fraction; site-specific cumulative dose	sterilization of occult microscopic disease with potential ICD	antigen release, dendritic-cell activation, cGAS-STING/type I interferon signaling	lymphopenia, organ toxicity, lymph-node damage depending on field size	PCI, ENI/RNI, selected high-risk anatomic regions	established in selected contexts
Whole-organ/large-field RT	variable; often conventional or low-intermediate dose	broad coverage of high-risk microscopic disease	may reduce occult disease burden across an organ or nodal basin	radiation-induced lymphopenia, marrow suppression, pneumonitis, impaired immune priming	whole-lung irradiation, extensive nodal fields, historical prophylactic strategies	highly context-dependent; requires strict risk-benefit selection
Ablative SBRT/SRS	usually >8 Gy/fraction	eradication of known gross lesions	strong local antigen release in selected settings	vascular damage, fibrosis, lymphocyte depletion, TREX1-associated immune attenuation at very high dose per fraction	oligometastatic or established lesions	therapeutic, not true prophylactic radiotherapy
Lymphocyte-sparing/organ-sparing planning	not a dose level; planning principle	preserve systemic immune competence while treating high-risk regions	reduces exposure of circulating blood, marrow, spleen, thymus, and draining lymph nodes	geographic miss if target reduction is excessive	modern PCI, selective ENI/RNI, ICI-combination settings	increasingly important but not yet standardized

cGAS-STING, cyclic GMP-AMP synthase-stimulator of interferon genes; ENI, elective nodal irradiation; ICD, immunogenic cell death; ICI, immune checkpoint inhibitor; LDRT, low-dose radiotherapy; MDSC, myeloid-derived suppressor cell; PCI, prophylactic cranial irradiation; PMN, pre-metastatic niche; RNI, regional nodal irradiation; RT, radiotherapy; SBRT, stereotactic body radiotherapy; SRS, stereotactic radiosurgery; TBI, total body irradiation; TREX1, three-prime repair exonuclease 1; Treg, regulatory T cell (note: “Gy” denotes gray).

## Tumor-type, metastatic-site, and clinical-scenario stratification

The concept of prophylactic RT should not be viewed as a one-size-fits-all strategy. Differences in metastatic patterns, organ-specific biology, therapeutic objectives, and toxicity tolerance necessitate disease-specific approaches. As an example, the marked neurotropism of SCLC provides a strong rationale for PCI, which has historical evidence supporting its use in carefully selected patients.^17^^,^^18^^,^^19^^,^^20^^,^^21^^,^^22^^,^^23^^,^^24^^,^^25^ By contrast, Ewing sarcoma or osteosarcoma typically disseminate to the lungs, rendering the pulmonary compartment a logical prophylactic target, albeit one limited by the risk of treatment-related pulmonary injury.^26^^,^^27^^,^^28^^,^^29^^,^^30^^,^^31^^,^^32^^,^^33^^,^^34^^,^^35^^,^^36^ NPC is characterized by predictable patterns of cervical lymphatic spread and high radiosensitivity, providing a strong rationale for risk-adapted ENI.^37^ However, in breast, cervical, esophageal, head-and-neck, and lung cancers, decisions regarding nodal irradiation must be individualized according to recurrence risk, systemic treatment options, anticipated survival benefit, and potential immunologic effects.^38^^,^^39^^,^^40^^,^^41^^,^^42^^,^^43^^,^^44^^,^^45^^,^^46^^,^^47^^,^^48^^,^^49^^,^^50^

Clinical context is another critical determinant of prophylactic RT decision-making. In patients receiving ICIs, unnecessary irradiation of draining lymph nodes or extensive lymphocyte-rich tissues may compromise antitumor immune responses.^38^^,^^39^^,^^49^^,^^50^ Alternatively, patients at substantial risk of sanctuary-site recurrence and with limited effective systemic treatment options may still derive benefit from prophylactic irradiation, even in the absence of definitive overall-survival advantages. Future clinical trials should avoid treating prophylactic RT as a uniform intervention across all settings. Instead, study designs should incorporate stratification according to tumor histology, patterns of metastatic spread, target-organ characteristics, concurrent systemic therapies, baseline immune competence, organ-at-risk limitations, projected survival, and biomarker-defined risk of occult metastatic disease.

## Safety, toxicity, and radiation-induced lymphopenia

Safety considerations are fundamental when evaluating the role of prophylactic RT. Because prophylactic treatment targets sites without clinically apparent disease or symptoms, the threshold for acceptable toxicity is considerably lower than that for RT directed at established microscopic tumors. Normal-tissue dose constraints, such as those outlined in QUANTEC, remain essential for assessing the risk of organ-specific toxicity. However, prophylactic RT introduces an additional consideration: any potential reduction in occult metastatic recurrence must outweigh the risk of treatment-related toxicity in patients who may never have developed disease within the irradiated region.

Treatment-related toxicity varies substantially according to the irradiated site, underscoring the need for individualized risk-benefit assessments in prophylactic RT. For example, neurocognitive impairment remains one of the major limitations of PCI, particularly among long-term survivors. Although hippocampal-sparing techniques and neuroprotective strategies can mitigate cognitive decline, they do not completely eliminate the potential adverse effects associated with brain irradiation. Evidence from the PREMER randomized phase III trial suggests that HA-PCI can mitigate early cognitive decline relative to conventional PCI. At 3 months, deterioration in delayed free recall was observed in 5.8% of patients treated with HA-PCI compared with 23.5% of those receiving standard PCI, while rates of BM, OS, and quality of life were comparable between groups.^25^ For PLI approaches, including WLI and PLI, the major toxicities include radiation pneumonitis, pulmonary fibrosis, restrictive ventilatory impairment, and potential long-term cardiopulmonary complications. These concerns are particularly important in patients with sarcoma, among whom WLI has been employed in selected clinical settings but is limited by the relatively low tolerance of normal lung tissue and the risk of chronic pulmonary toxicity.^34^ Although advanced techniques such as proton therapy and IMPT may reduce radiation exposure to the heart, breasts, and other adjacent normal tissues, long-term outcome and safety data remain limited.^35^^,^^36^ For ENI or regional nodal RT, the spectrum of treatment-related toxicity varies according to the anatomical site being treated. Potential adverse effects include injury to the salivary glands, thyroid, lungs, heart, bowel, bone marrow, and lymphatic system, as well as disruption of immune functions mediated by draining lymph nodes.^38^^,^^39^^,^^49^^,^^50^ In breast cancer, the MA.20 trial showed that the addition of RNI improved disease-control outcomes but did not confer a significant overall-survival advantage. This benefit was accompanied by increased rates of grade ≥2 acute pneumonitis (1.2% vs. 0.2%) and lymphedema (8.4% vs. 4.5%).^40^ RIL is also increasingly recognized as an important systemic adverse effect of RT. Recent analyses indicate that absolute lymphocyte counts can decline to a median of approximately 24% of pretreatment levels by completion of intracorporeal RT, with recovery remaining incomplete at roughly 55% of baseline after one year.^65^ These findings highlight the need for prophylactic RT studies to incorporate comprehensive safety assessment, including neurocognitive testing, pulmonary-function evaluation, lymphedema surveillance, immune-cell monitoring, and patient-reported outcomes, rather than considering toxicity solely as a technical treatment parameter.

RIL has become an increasingly important consideration in the era of cancer immunotherapy. Because lymphocytes are highly radiosensitive, clinically significant depletion may occur even at relatively low radiation doses.^13^ The likelihood and severity of RIL depend not only on the prescribed dose but also on factors such as treatment volume, fractionation schedule, radiation exposure to circulating blood, changes in absolute lymphocyte counts over time, and irradiation of lymphocyte-rich structures, such as lymph nodes, bone marrow, spleen, lungs, heart, and major blood vessels.^13^^,^^65^ From a clinical perspective, RIL may impair immune surveillance, diminish systemic antitumor immune responses, and potentially reduce the therapeutic effectiveness of ICIs.^13^^,^^65^ Emerging evidence further indicates that cranial irradiation may contribute to lymphocyte depletion and poorer clinical outcomes in patients with BMs, highlighting that even localized RT may exert broader systemic immunological effects.^66^

These concerns should not be interpreted as arguments against the use of prophylactic RT. Rather, they underscore the importance of careful patient selection and thoughtful treatment design. Future protocols should prioritize minimization of elective treatment volumes when oncologically appropriate, incorporation of hippocampal and organ-at-risk sparing techniques, adherence to lung and bone marrow dose constraints, implementation of lymphocyte-sparing strategies, and optimization of treatment sequencing with systemic therapies and immunotherapeutic agents. Future prospective studies should incorporate comprehensive immune profiling, including baseline and longitudinal assessment of immune status, monitoring of absolute lymphocyte count dynamics, characterization of lymphocyte subsets, and evaluation of the relationships between immune-related toxicity, metastatic recurrence, and survival outcomes. Within this framework, safety should be regarded as a fundamental biological and clinical endpoint rather than a secondary technical consideration.

## Biomarkers and surveillance in the asymptomatic prophylactic setting

The use of biomarkers to guide prophylactic RT is an appealing concept, although it has not yet been validated for routine clinical practice. Potential biomarker candidates include circulating tumor DNA (ctDNA), the quantity and biological characteristics of CTCs, extracellular-vesicle content and organ-specific integrin signatures, inflammatory cytokine profiles, MDSC- and Treg-associated immune signatures, tissue-based immune biomarkers, radiomic features, and functional imaging measures of hypoxia, perfusion, metabolism, or immune activity.^67^^,^^68^^,^^69^^,^^70^

Biomarker development is particularly challenging in the asymptomatic prophylactic setting, where disease burden is substantially lower than in patients with established malignancy. Intermittent ctDNA shedding, the rarity of detectable CTCs, technical variability in extracellular-vesicle analyses, and the limited ability of current imaging modalities to visualize microscopic PMN alterations all complicate reliable risk assessment and surveillance.^67^^,^^68^^,^^69^^,^^70^ False-positive biomarker or imaging findings may result in unwarranted irradiation of normal tissues, while false-negative results risk postponing treatment in patients with occult metastatic spread. At present, evidence-based thresholds for initiating RT, adjusting target volumes, selecting dose, or integrating prophylactic RT with systemic therapy have not been established.

Therefore, at present, biomarkers are best suited for clinical trial enrichment and mechanistic correlative analyses rather than routine clinical decision-making. Future prophylactic RT trials should prospectively incorporate longitudinal assessment of ctDNA and CTCs, extracellular-vesicle signatures, immune-cell profiling, cytokine panels, and imaging biomarkers, ideally with predefined intervention thresholds. Such studies should evaluate whether biomarker-defined high-risk states can reliably identify patients who derive benefit from prophylactic irradiation, and whether post-RT biomarker dynamics correlate with PMN remodeling, metastatic outcomes, immune-related toxicity, and survival.

## Integration with immunotherapy and other systemic agents

Although biologically attractive, the integration of prophylactic RT or LDRT with immunotherapy has not yet been clinically defined. RT can increase antigen availability, trigger immunogenic cell death, activate cGAS-STING/type I interferon signaling, enhance dendritic-cell priming, and support T cell infiltration, forming the mechanistic basis for its potential synergy with ICIs.^8^^,^^10^^,^^58^^,^^67^ However, these same radiobiological effects may also result in systemic immune suppression, including lymphocyte depletion, disruption of draining lymph-node integrity, impaired antigen presentation, and overall reduction in immune fitness.^13^^,^^38^^,^^39^^,^^49^^,^^50^^,^^59^^,^^71^ Accordingly, in the prophylactic setting, RT should not be combined with ICIs empirically. The therapeutic balance is likely influenced by dose per fraction, total dose, irradiated volume, treatment timing, anatomical site, and inclusion of lymphoid structures within the radiation field. This issue is particularly important in the context of ENI, as draining lymph nodes serve not only as potential reservoirs of occult metastatic disease but also as critical hubs for dendritic-cell migration and antitumor T cell activation.^38^^,^^39^^,^^49^^,^^50^^,^^72^ Approaches that limit treatment volume or spare lymphocyte-rich tissues may help preserve immune function, whereas extensive nodal irradiation or suboptimal treatment timing could weaken systemic antitumor immunity and diminish the effectiveness of ICIs.^38^^,^^39^^,^^49^^,^^50^^,^^59^

The optimal timing and sequencing of RT relative to systemic therapy have yet to be clearly defined. In some settings, concurrent administration of RT and ICIs may promote tumor antigen release and enhance recruitment of effector immune cells, whereas sequential approaches may allow immune priming to occur before irradiation or facilitate lymphocyte recovery prior to ICI treatment.^71^ Beyond ICIs, prophylactic RT and LDRT may be combined with a variety of systemic therapies, including chemotherapy, anti-angiogenic agents, targeted therapies, DNA damage response inhibitors, cytokine-based treatments, cancer vaccines, and adoptive cellular therapies. While these combinations may enhance tumor control through improved cytotoxicity, vascular remodeling, immune-cell trafficking, or antigen presentation, they may also increase the risk of bone marrow suppression, vascular complications, normal-tissue toxicity, and immune-cell depletion.^60^^,^^73^^,^^74^ For instance, preclinical studies on hepatocellular carcinoma have demonstrated that combining LDRT with PD-L1 and VEGFA inhibition can enhance CD8^+^ T-cell-driven antitumor immunity. These findings support the concept that LDRT may function primarily as an immune-modulating and sensitizing strategy rather than solely as a direct cytotoxic intervention.^73^ Future clinical trials should incorporate comprehensive translational endpoints, including longitudinal assessment of lymphocyte dynamics, immune-cell subsets, T cell receptor clonality, myeloid-cell polarization, type I interferon signaling, ctDNA clearance, organ-specific PMN biomarkers, and radiation exposure to circulating blood, bone marrow, spleen, and draining lymph nodes. Such measures are essential to clarify whether prophylactic RT primarily enhances antitumor immunity, remodels PMNs, or inadvertently suppresses systemic immune responses.^13^^,^^59^^,^^71^^,^^72^^,^^73^

## Controversies, unresolved questions, and barriers to clinical translation

Despite growing enthusiasm for prophylactic RT, its clinical value remains the subject of ongoing debate. A key unresolved issue is whether reductions in regional recurrence or organ-specific metastatic failure are sufficient endpoints to warrant treatment when corresponding improvements in OS are lacking, inconsistent, or obscured by effective salvage interventions. This question carries particular weight in the prophylactic setting, where radiation is directed at disease-free or asymptomatic regions, and the acceptable threshold for toxicity is necessarily lower than that for gross tumor treatment. A second unresolved issue concerns the immunological impact of large-field prophylactic irradiation in the era of immunotherapy. While elective irradiation may eliminate occult tumor deposits, it may also expose circulating lymphocytes, bone marrow, lymph nodes, the spleen, or other immune-related tissues to radiation, potentially compromising systemic immune surveillance and diminishing the effectiveness of ICIs.^13^^,^^38^^,^^39^^,^^49^^,^^50^^,^^59^^,^^65^^,^^75^ A third area of uncertainty involves the clinical utility of PMN-directed LDRT. Although preclinical and early translational evidence suggests that LDRT may modulate macrophage phenotypes, reduce suppressive myeloid and regulatory T cell populations, improve vascular function, and render metastatic niches less supportive of tumor growth, these observations have not yet been validated in clinical practice.^11^^,^^12^^,^^58^ However, emerging preclinical evidence indicates that exposure to subtherapeutic radiation doses may, under certain conditions, remodel the pre-metastatic lung niche in ways that facilitate metastatic colonization and growth. These findings underscore the importance of validating optimal dose parameters and treatment timing in an organ-specific manner before clinical implementation.^76^

Several practical challenges continue to hinder clinical translation. Earlier studies of PLI were often limited by small cohorts, heterogeneous patient populations, outdated systemic treatment approaches, variable imaging follow-up protocols, and insufficient assessment of immune-related outcomes.^26^^,^^27^^,^^28^^,^^29^^,^^30^^,^^31^^,^^32^^,^^33^^,^^34^^,^^35^^,^^36^ More broadly, future investigations must resolve key uncertainties regarding optimal dose and fractionation schedule, integration with systemic therapies, organ-specific toxicity profiles, patient-selection criteria, and the most appropriate clinical endpoints. Preventive clinical trials are inherently challenging because they often require large patient cohorts and extended follow-up periods to capture meaningful outcomes. However, if prospectively validated, measures such as metastasis-free survival, ctDNA dynamics, immune-related biomarkers, patient-reported outcomes, quality-of-life assessments, and organ-specific imaging findings may serve as useful surrogate or intermediate endpoints.^67^^,^^68^^,^^69^^,^^70^^,^^77^ Ethical trial design should incorporate clear risk-benefit communication, strict adherence to dose constraints, lymphocyte-sparing planning strategies, systematic patient-reported toxicity evaluation, quality-of-life monitoring, and predefined stopping rules for excess toxicity. Ultimately, the central translational question is not whether prophylactic RT can reduce occult disease in selected contexts, but whether it can achieve this with an acceptable therapeutic ratio in the contemporary era of advanced imaging, immunotherapy, and biomarker-guided care.

## Future directions: flash RT, spatial omics, artificial intelligence, and precision prevention

The next phase of prophylactic RT should move away from wide, non-selective irradiation toward biologically guided precision prevention. This approach depends on accurately identifying patients at high risk of occult spread, characterizing the specific organ or nodal microenvironment that supports disease propagation, and tailoring dose regimens to achieve defined biological effects. Equally important is the integration of RT with systemic therapies in a way that maintains immune function while enhancing overall therapeutic efficacy.

Spatial transcriptomics, single-cell sequencing, multiplex imaging platforms, and circulating biomarker analyses may offer powerful tools to define PMN biology and to identify patients who are most likely to benefit from preventive strategies. In particular, liquid biopsy approaches, particularly ctDNA kinetics, may enable refined risk stratification, early evaluation of therapeutic response, and sensitive detection of minimal residual disease.^67^^,^^68^ Concurrently, spatial omics and single-cell technologies may delineate organ-specific immune, stromal, vascular, and extracellular vesicle signaling programs that shape the PMN.^56^^,^^57^^,^^78^^,^^79^^,^^80^ In parallel, artificial intelligence-driven integration of multimodal datasets, including imaging, histopathology, genomics, radiomics, dosimetry, and circulating biomarkers, may enhance prediction of metastatic tropism, therapeutic response, normal tissue susceptibility, and patient-specific target definition.^81^^,^^82^ Collectively, these technologies may shift prophylactic RT away from an anatomy-driven paradigm grounded in historical patterns of failure toward a dynamic, biologically adaptive strategy guided by patient- and tumor-specific determinants.

FLASH RT is an additional emerging strategy of interest. By administering radiation at ultra-high dose rates, it has demonstrated in preclinical and early translation studies the potential to limit normal tissue toxicity while preserving antitumor efficacy.^83^^,^^84^^,^^85^ This feature is particularly appealing in prophylactic contexts, where the treated regions are clinically uninvolved and preservation of normal tissue is paramount. Nevertheless, FLASH RT should not be considered equivalent to LDRT, as the two approaches differ fundamentally in both dose delivery and underlying biological intent. The underlying biological mechanisms, immunological consequences, dose-rate relationships, technical constraints, and feasibility in large or anatomically complex prophylactic treatment fields remain incompletely understood.^83^^,^^84^^,^^86^^,^^87^ Accordingly, FLASH RT should currently be regarded as an emerging investigational approach rather than an established clinical strategy for prophylactic RT.

Future investigations should be guided by biomarker-informed and mechanistically grounded designs, incorporating patient stratification based on tumor histology, patterns of metastatic spread, involved target organs, concurrent systemic therapies, immune context, and molecular indicators of occult disease risk. Trial endpoints should extend beyond metastasis-free and OS to incorporate functional and immunobiological outcomes, including neurocognitive performance, pulmonary function, quality of life, radiation-associated lymphopenia, lymphocyte subset dynamics, T cell receptor clonality, myeloid polarization patterns, ctDNA kinetics, and organ-specific PMN markers. These comprehensive endpoints will be critical to determine whether prophylactic RT can safely reduce metastatic risk, reprogram the metastatic microenvironment, or enhance the efficacy of systemic therapies.

## Conclusion

Prophylactic RT should be viewed as a context-specific intervention rather than a blanket strategy for preventing metastatic disease. In carefully selected indications such as PCI or risk-adapted ENI/RNI, it may reduce regional or site-specific relapse, although improvements in OS are inconsistent and treatment-related toxicity remains a key limitation. Its future development is therefore less about expanding elective irradiation and more about refining patient selection, improving target delineation, minimizing immune and lymphocyte damage, and integrating biomarker-driven approaches alongside systemic therapies.

LDRT-driven modulation of immune and stromal compartments presents an intriguing hypothesis for targeting metastasis at the micrometastatic or PMN level. Nevertheless, this approach is still experimental and should not be regarded as a validated component of routine clinical care. Well-designed prospective studies are required to establish the optimal dosing strategy, target sites, treatment timing, immunological impact, and appropriate patient selection. Moving forward, a balanced framework that integrates local cytotoxic control, preservation of immune function, organ-specific tolerance, tumor-associated metastatic risk, and coordination with systemic therapy will be essential for transforming prophylactic RT from a primarily anatomically based practice into a precision-guided approach to metastasis prevention.

## Acknowledgments

This work was supported by the Henan Youth and Middle-aged Health Science and Technology Innovation Leaders Training Project (no. YXKC2022004), the Henan Health Young and Middle-aged Discipline Leader Project (no. HNSWJW-2022011), the Henan Medical Science and Technology Tackling Programme Provincial-Ministry Joint Major Project (no. SBGJ202401004), and the Key Research and Development Projects of Henan Province (no. 251111310100). We appreciate the linguistic assistance provided by TopEdit (www.topeditsci.com) during the preparation of this manuscript. Additionally, Figures 1, 2, and 3 were created with BioRender.com under a valid publication license (Copyright 2026).

## Author contributions

Conceptualization, J.L., Y.L., and Z.S.; methodology, Q.W. and Z.L.; investigation, X.Z., L.L., and Y.Y.; writing – original draft, J.L.; writing – review and editing, Q.W., Z.S., and Y.L.; funding acquisition, Y.L.

## Declaration of interests

The authors declare no competing interests.
